# Nb, F-codoped TiO_2_ hollow spheres with high visible light photocatalytic activity

**DOI:** 10.1186/1556-276X-8-508

**Published:** 2013-12-04

**Authors:** Mingqi Gao, Youlong Xu, Yang Bai, Fang Xiao

**Affiliations:** 1 Electronic Materials Research Laboratory, Key Laboratory of the Ministry of Education & International Center for Dielectric Research, Xi'an Jiaotong University, Xi'an 710049, China

**Keywords:** Photocatalytic activity, Sol–gel preparation, Nb, F-codoping, TiO_2_, Solar energy materials, Hollow sphere

## Abstract

Nb, F-codoped TiO_2_ hollow spheres (NFTSs) were successfully synthesized via a hydrothermal method with niobium oxide, hydrofluoric acid, and tetrabutyl titanate. The obtained spheres were hollow, with a diameter of about 2 μm, and the sphere wall was made up of nanorods arranged close together. The NFTSs presented anatase phase with more {001} facets exposed, which could be mainly attributed to F ions which were preferentially adsorbed on the {001} facets during the crystal growth process. Ti^3+^ states in NFTSs were increased due to Nb, F-codoping, resulting in the decrease of the band gap and the red shift of the absorption edge of the NFTSs. The NFTSs exhibited 20.1% higher photocatalytic speed compared to P25 on the degradation of methylene orange.

## Background

Among various oxide semiconductor photocatalysts, TiO_2_ has been studied extensively and considered to be the most appropriate for applications in the environmental field because of its biological and chemical inertness, cost effectiveness, strong oxidizing power, and long-term stability against chemical corrosion and photocorrosion [1]. The photocatalytic ability of TiO_2_ is strongly affected by morphologies, phase structures, macroscopic structures, and so on [2-10]. In addition, the surface property is a key factor influencing the photocatalytic activity [2]. The surface density for the {001} facets has been demonstrated to be higher than that for other facets with undercoordinated Ti atoms [11]. The exposed {001} facets of anatase TiO_2_ have been proven to possess high surface energy, which induces high reactivity [12,13]. Therefore, photocatalysts with higher reactivity can be obtained by controlling the exposed crystal facets of TiO_2_[14].

Moreover, to improve the photocatalytic activity of titania, reducing the band gap has been proven as a valid approach. An effective way to red shift the absorption edge can be achieved by doping one kind of element, such as F, Nb, and Mn [15-18]. After doping, Ti^3+^ states in TiO_2_ increase effectively. The existence of Ti^3+^ plays an important role on the enhancement of the photocatalytic activity [15]. Thus, the use of codoping by two or more elements is reported to result in a significant improvement for increasing the photocatalytic activity, such as Nb and N [19,20], Zr and Y [21], and F, B, and Si [22]. In our previous work, titania micron beads codoped with Nb and F were synthesized and used in DSSCs with beneficial result [23].

In this paper, the Nb, F-codoped TiO_2_ hollow spheres (NFTSs) were synthesized via a facile hydrothermal process using niobium oxide and hydrofluoric acid as codoping source. The phase structure, morphology, chemical composition, band gap energy, and photocatalytic activity of the obtained product were investigated.

## Methods

### Preparation of NFTSs

All chemicals, including tetrabutyl titanate, niobium oxide (Nb_2_O_5_) powder, hydrofluoric acid, and lactic acid, were of analytical grade and used as received without further purification.

Nb_2_O_5_ was dissolved using hydrofluoric acid to obtain a clear and transparent solution. The Ti precursor was prepared using tetrabutyl titanate chelated with lactic acid. Then, the solution was dropwise added into the Ti precursor. Finally, deionized water was added to obtain a clear aqueous sol precursor, including Ti^4+^, Nb^5+^, and F^−^ with concentrations of 0.5, 0.01, and 5.0 M, respectively.

The sol precursor was transferred into a Teflon autoclave and then heated at 110°C for 20 h, followed with 20 h at 180°C in the furnace. The resulting precipitates were filtrated, centrifuged and washed with deionized water and alcohol, and then dried at 50°C overnight in an oven.

### Characterization of the NFTSs

The phase identification and crystal structure of the samples were measured by powder X-ray diffraction (XRD, X'pert PRO, PANalaytical, Holland, The Netherlands) with a monochromatized source of Cu Kα1. The sample morphology was characterized with a field-emission scanning electron microscope (SEM, JEM-6700 F, JEOL Ltd., Tokyo, Japan) and a transmission electron microscope (TEM, JEM-2100, JEOL Ltd., Tokyo, Japan). The chemical composition of the sample was recorded by X-ray photoelectron spectroscopy (XPS, AXIS-Ultra DLD, Kratos Analytical Ltd., Manchester, England) with a monochromatized Al Kα X-ray source. UV-visible diffusion reflectance spectroscopy measurements were carried out on a U-4100 spectrophotometer (Hitachi Co., Tokyo, Japan) equipped with a diffuse reflectance integration sphere attachment.

### Photocatalytic activity measurements

Photoirradiation was carried out with a 300-W Xe arc lamp fitted with an AM 1.5G filter to give a simulated light irradiance with an intensity of 100 mW cm^−2^. Photocatalytic activity was evaluated by the photodegradation of methyl orange (MO), whose initial concentration was 20 mg L^−1^. Before irradiation, the suspensions (0.1 g L^−1^) were ultrasonically dispersed in the dark for 60 min to ensure adsorption equilibrium. After irradiation, the absorbance of the MO solution was measured at regular intervals with a UV-vis spectrophotometer (UV-3300PC, Mapada, Shanghai, China).

## Results and discussion

**Figure 1:**
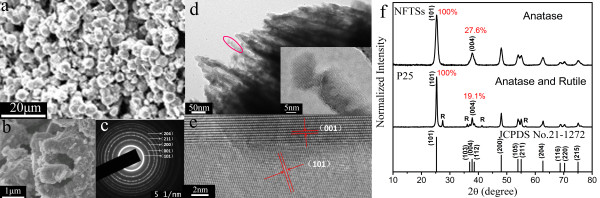
**The morphology and structure characterization of NFTSs. (a)** SEM image, **(b)** a magnification of the SEM image of typical broken hollow spheres, **(c)** SAED image, **(d)** TEM images, **(e)** HRTEM image, and **(f)** XRD patterns of the NFTS sample.

The SEM image of the NFTSs is displayed in Figure 1a. The hollow sphere structure is further corroborated by the corresponding SEM image (Figure 1b), which displays some broken ones. As shown, the outside diameter of the spheres is above 2 μm, while the inner diameter of the hollow section is about 1 μm. In the TEM image (Figure 1d), a number of nanorods with an average width of 20~30 nm and length of about 0.5 μm were arranged close together to form the sphere wall.

The NFTSs can be defined as anatase by the selected area electron diffraction (SAED) image (Figure 1c). Figure 1f shows the normalized XRD pattern of the as-prepared NFTSs and P25. The peaks of the former can be accurately attributed to anatase TiO_2_ according to JCPDS no. 21-1272 without any other phase. The relative ratio of the diffraction peak (004) of NFTSs (27.6%) based on integration of area is higher compared to that of P25 (19.1%). This demonstrates that the {001} facets for the NFTSs have been enhanced.

As known [2,14,24], the surface energy and reactivity of the {001} facet are relative higher than those of other facets in the anatase TiO_2_. During the process of TiO_2_ crystal growth, fluorine ions in the sol precursor were preferentially adsorbed on the {001} facets, which retarded the growth and facilitated the formation of {001} facets. As shown in the high-resolution transmission electron microscopy (HRTEM) image (Figure 1e), the crystal faces paralleling to the top and bottom of the nanorods are {001} facets. Therefore, the XRD result displays that more {001} facets are exposed in NFTS sample, which implies better photocatalytic reactivity.

**Figure 2:**
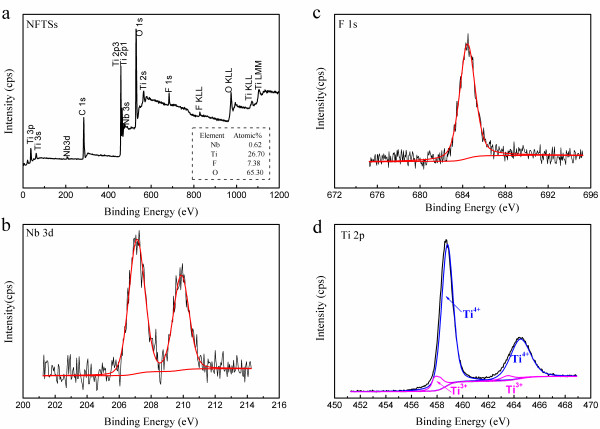
**XPS spectra of NFTSs. (a)** Survey spectrum, **(b)** Nb 3*d* spectrum, **(c)** F 1*s* spectrum, and **(d)** Ti 2*p* spectrum of the NFTS sample.

The XPS spectra of the NFTS sample are illustrated in Figure 2. The XPS spectra show obvious Nb 3*d* and F 1*s* peaks at about 207 and 685 eV, respectively. For the Ti 2*p*3/2 peak, the binding energy of Ti^3+^ (457.8 eV) [25] is lower than that of Ti^4+^ (458.8 eV) [26]. The shape and position of the Ti peaks can be assigned as a mixture of Ti^4+^ and Ti^3+^ states, as shown in Figure 2d. The generation of the Ti^3+^ states is due to the introduction of Nb and F [15,20]. The existence of Ti^3+^ centers in TiO_2_ enhances the photocatalytic activity of the sample [15].

**Figure 3:**
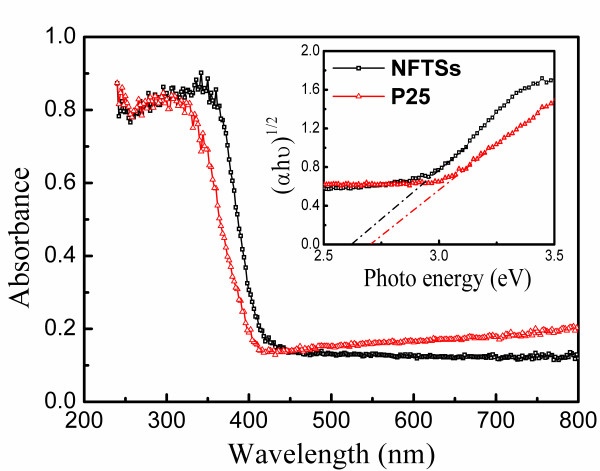
**UV-visible diffusion reflectance spectra of the NFTSs and P25.** Inset: plots of (*αhν*)^1/2^ versus photon energy (*hν*).

In Figure 3, the UV-visible diffusion reflectance spectrum of the anatase NFTSs shows an obvious red shift in the absorption edge compared with P25. This result clearly directs a decrease in the band gap energy (*E*_g_) of NFTSs, which can be obtained from a plot of (*αhν*)^1/2^ versus photon energy (*hν*). The narrower band gap could cause a lower oxidation power of the photoinduced holes [2], which suggests higher photocatalytic activity.

**Figure 4:**
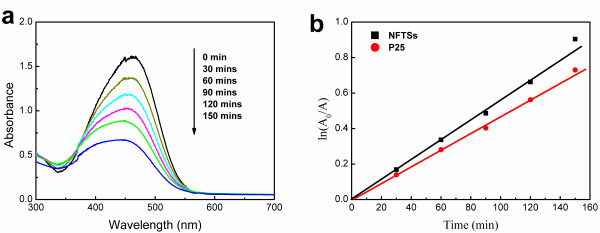
**Photocatalytic spectra of NFTSs and P25. (a)** Absorption spectra of MO at various photocatalysis treatment times by NFTSs. **(b)** Plots of ln(*A*_0_/*A*) versus time for NFTSs and P25.

The absorption peak of the MO solution appears at 467 nm, as shown in Figure 4a. With the time prolongation of irradiation, the peak value declines rapidly due to NFTSs. To evaluate the photocatalytic activities of the NFTSs and P25 on degradation of MO, the functions of ln(*A*_0_/*A*) versus time are plotted in Figure 4b, where *A* denotes the absorption of MO changing with illumination time and *A*_0_ the initial absorption at 467 nm. The plots are linear, and the slope *k* can represent the photocatalytic speed (min^−1^) of the powder. The NFTSs (*k*_NFTSs_ = 5.61 × 10^−3^) show 20.1% higher photocatalytic speed than P25 (*k*_P25_ = 4.67 × 10^−3^). The higher photocatalytic performance of the NFTSs can be ascribed to the more exposed {001} facets with higher reactivity, increased Ti^3+^ states, and decreased band gap caused by Nb, F-codoping and the unique hollow structure, which allows multi-reflections of electromagnetic waves to absorb more incident light [8,9] and provides more active reaction sites. Additionally, the NFTSs can be readily separated from the suspension by sedimentation and filtration after photocatalytic reaction, which are obviously superior to P25. Consequently, the NFTSs possess a favorable photocatalytic activity on the degradation of MO.

## Conclusions

In summary, the anatase NFTSs with more {001} facets exposed and lower band gap energy were successfully prepared using a facile hydrothermal method though Nb, F-codoping. The prepared NFTSs were proven to possess 20.1% higher photocatalytic speed than P25 on the degradation of MO. The NFTSs demonstrate a favorable photocatalytic activity, and they are expected to find extended applications in environment and solar energy fields.

## Competing interests

The authors declare that they have no competing interests.

## Authors' contributions

MQG and YLX designed the experiments. MQG, YB, and FX carried out the experiments and performed data analysis. MQG wrote the paper. All authors read and approved the final manuscript.
